# Beyond interpersonal bonds: linking leader-member exchange and coworker exchange on flourishing and the mediating role of thriving at work

**DOI:** 10.3389/fpsyg.2026.1812214

**Published:** 2026-08-05

**Authors:** Sevgi Elmas Atay, Merve Kırbaşlar, Caner Günaydın, Engin Karaman

**Affiliations:** 1 School of Business, Human Resource Management Department, Istanbul University, Istanbul, Türkiye; 2 Hereke Omer Ismet Uzunyol Vocational School, Human Resource Management Program, Kocaeli Universitesi, Körfez, Türkiye; 3 Faculty of Economics, Administrative and Social Sciences Department of Data Science and Analytics, Istanbul Topkapi Universitesi, İstanbul, Türkiye

**Keywords:** academics, coworker exchange, flourishing, leader-member exchange, thriving

## Abstract

**Introduction:**

Positive psychology research emphasizes the concept of flourishing as one of the primary goals in human life. Supporting academics’ professional growth and wellbeing is crucial because it affects their students’ educational experiences, the efficiency of their institutions, and the overall state of the national research ecosystem. Drawing on social exchange and self-determination theories, this study examined how two forms of interaction at work, leader-member exchange (LMX) and coworker exchange (CWX), influence flourishing and the mediating role of thriving at work in this relationship.

**Methods:**

The data were collected from 418 academics employed at public and private universities in Turkey. Leader–member exchange and coworker exchange were modeled as the predictors, thriving at work was specified as the mediator, and flourishing was examined as the outcome variable. Structural equation modeling was used to test the proposed relationships.

**Results:**

Structural equation modeling revealed that thriving mediated the relationship between leader-member exchange and flourishing. Furthermore, coworker exchange demonstrated a direct correlation with flourishing, whereas leader-member exchange was associated with flourishing only indirectly through thriving, indicating that the quality of interactions with leaders and colleagues corresponded to distinct levels of wellbeing among academics.

**Conclusion:**

These findings highlight the distinct roles of leader and coworker exchanges in terms of shaping flourishing among academics. The study offers beneficial implications for promoting psychological functioning at research-oriented institutions while also advancing theoretical knowledge of exchange-based wellbeing.

## 1 Introduction

Positive psychology has emerged as a prominent discipline within psychology, moving the emphasis from treating disease and dysfunction alone to fostering wellbeing and flourishing (Huppert and So, 2013; Seligman, 2011). Flourishing encompasses both hedonic and eudaimonic aspects and is considered a more comprehensive approach to evaluating individuals’ wellbeing than the subjective measure of wellbeing typically referred to as happiness. This holistic perspective is especially significant in organizational settings due to the extensive effects of psychological distress on individuals, groups, and organizational levels (Singh et al., 2019). In this regard, social exchange theory (SET) provides important insights into how workplace interactions, such as leader-member exchange (LMX) and coworker exchange (CWX), influence employees’ wellbeing. SET emphasizes that expectations of reciprocity play an essential role in individuals’ actions (Blau, 1964). Thus, the presence of supportive coworkers and a competent leader is a crucial component of a productive work environment. Employees who engage in high-quality interactions with their supervisor and those who engage in low-quality interactions might display reluctance to collaborate, leading to intergroup disputes. Therefore, the interaction between a leader and an employee is equally significant as that between coworkers (Sherony and Green, 2002). It is well established that supportive colleague connections are crucial in acquiring new information and skills in the workplace (Carmeli et al., 2009), and high-quality LMX creates a working atmosphere characterized by mutual connection, trust, and respect between employees and their leader (Martin et al., 2018). Given that interactions at work are social resources that affect flourishing, LMX and CWX may be essential for understanding how these social interactions influence an individual’s personal development at work and overall wellbeing.

The extensive impact of social interactions, including LMX and CWX, on human functioning could operate through indirect mechanisms. Based on the idea that social exchange at work is a potential resource for increasing learning and vitality, Walumbwa et al. (2020) showed that LMX and coworkers’ support affect thriving at work. In this sense, self-determination theory (SDT) can explain how social exchange and personal growth at work are related to focusing on needs that are fundamental requirements for healthy human development. SDT argues that people are innately proactive, intrinsically motivated, and directed toward natural development through an integrative process. It prioritizes the significance of fulfilling psychological needs and having self-driven motivation for personal growth and development (Deci and Ryan, 2012; Graves and Luciano, 2013). This study is primarily grounded in SET in order to explain how high-quality relationships with leaders and coworkers provide essential resources that contribute to flourishing, while SDT is used to explain the mediating role of thriving at work.

A sense of continual growth and development at work is crucial for navigating unpredictable situations and maintaining productivity and wellbeing, particularly in today’s dynamic business environment, where employees are continuously required to respond to emerging challenges (Porath et al., 2012). In organizational contexts, meeting these demands is associated with the concept of thriving. Thriving, defined by the experience of vitality and learning, has the capacity to enhance flourishing, which is focused on developmental progression and productivity. Therefore, by fostering positive social exchanges, such as LMX and CWX, organizations can enhance thriving at work while also supporting their employees in successfully adapting to changing environments, ensuring sustained performance and resilience throughout their careers. High-quality LMX, characterized by trust, respect, and mutual commitment, provides workers with the tools and support needed to engage in proactive behaviors crucial to fostering creativity and exceeding performance standards. Similarly, positive CWX develops a culture of support and collaboration among colleagues, encouraging information sharing, mutual help, and a collective ability to adapt and sustain productivity and wellbeing. Thus, social interactions constitute a significant component of human requirements. Blustein et al. (2023) suggested that organizations should invest in positive work relationships to support social contribution and self-determination needs, thereby providing more meaningful work experiences. Beyond the professional setting, work interactions hold the capacity to provide insightful viewpoints that could benefit all aspects of life and ultimately enhance overall life satisfaction (Colbert et al., 2016).

Given the importance of interpersonal relationships for employee flourishing, academic environments constitute a particularly relevant context for examining these dynamics. This context is especially important in developing economies, where higher education institutions serve as an intermediary between education, research, and work, and where academics function not merely as instructors but as pivotal figures linking theory to practice and enabling technology transfer within the research ecosystem. Analyzing these interactions among academics enhances the understanding of how relationship resources support psychological stimulation and overall wellbeing. In addition, this research enhances the cultural and institutional diversity represented in the organizational psychology literature by focusing on a developing country. Workplace interactions may differ depending on socioeconomic and cultural conditions, particularly in environments where relational interdependence is significant. Thus, evidence from Turkey provides context-specific insights into how interaction dynamics promote emerging research ecosystems, enabling an evaluation of the relevance of thriving at work in various institutional contexts.

Despite considerable theoretical support for LMX and CWX as key relational resources, there remains a significant gap in understanding how these specific social exchanges contribute to flourishing. Although LMX is one of the most established concepts in leadership research, recent studies suggest that the LMX literature continues to evolve, with increasing attention to alternative conceptualizations of exchange relationships (Fein and Tziner, 2021). In particular, Andersen et al. (2020) argue that contemporary LMX research should more closely align its conceptualization and measurement with its theoretical foundations, calling for greater consistency between theory and empirical research. Consistent with this perspective, Liu J. et al. (2021) examined LMX and CWX simultaneously, highlighting the importance of considering workplace exchange relationships more broadly rather than focusing solely on the leader-follower dyad. Building on these developments, the present study extends this line of research by investigating how leader-member exchange and coworker exchange jointly shape employee flourishing through the mediating role of thriving at work. As previously mentioned, relational resources, defined as intangible assets created through social interactions in the work environment, are critical for promoting employee wellbeing and productivity (Spreitzer et al., 2005). Researchers have investigated the relationship between LMX and CWX, as well as the positive workplace outcomes. However, these studies hardly provide a thorough examination of the specific processes by which relational resources improve thriving at work and provide a more comprehensive measure of employees’ wellbeing. While individual antecedents of thriving have received significant attention, recent studies have highlighted the importance of considering multiple workplace exchange relationships when explaining employee outcomes (Liu J. et al., 2021; Tang et al., 2022). Some studies considered LMX and coworker support as relational resources (Walumbwa et al., 2020; Zhai et al., 2020); however, they often focused on these characteristics in isolation, neglecting their combined impact on employee outcomes. According to SET, people engage in social interactions with the expectation of receiving benefits in exchange for their contributions (Takeuchi et al., 2011). Nevertheless, the combined influence of LMX and CWX on flourishing and the role of thriving at work in this relationship have not yet been fully examined. Shedding light on this neglected aspect could provide a more comprehensive understanding of the quality, reciprocity, and relational depth that characterize workplace relationships. Hence, this study contributes to the workplace relationship literature by shifting attention from narrowly defined work-related attitudes to broader forms of employee functioning. To our knowledge, it is among the first studies to examine LMX and CWX jointly as antecedents of flourishing, with thriving at work serving as the underlying mechanism.

This study contributes to the literature mainly in three ways. First, whereas LMX and coworker resources have largely been examined separately, leaving their interrelationship and relative effects unresolved (Walumbwa et al., 2020), this study models them simultaneously, thereby disentangling their distinct relational pathways to flourishing. Second, it conceptualizes thriving at work as a developmental mechanism that translates relational resources into a broader, life-level outcome (Porath et al., 2012; Spreitzer et al., 2005), rather than treating it merely as a predictor of work-specific outcomes. Third, the study adopts flourishing as its focal outcome instead of a domain-specific work attitude or behavior. As a comprehensive indicator of psychological functioning that integrates hedonic and eudaimonic wellbeing (Diener et al., 2010; Huppert and So, 2013), flourishing captures how the benefits of high-quality workplace relationships extend beyond the workplace (Colbert et al., 2016).

Furthermore, the proposed model is examined within a non-Western, knowledge-intensive academic context, enabling an assessment of whether these relational mechanisms generalize beyond the predominantly Western corporate settings in which they have been established. In doing so, the study extends SET by clarifying how workplace social exchange processes translate into flourishing and offers practical insights into how universities can foster academics’ development and wellbeing through high-quality workplace relationships.

## 2 Theoretical background and hypotheses

### 2.1 Turkish context

Turkey is widely acknowledged as a bridge between two distinct cultures, spanning Europe and Asia. In a developing country like Turkey, academics often face substandard working conditions compared with global standards. A growing body of research indicates that academics in Turkey are regularly subjected to heavy workloads, high job insecurity, and limited income (Tayfur Ekmekci et al., 2021; Toker, 2011), which could negatively affect their flourishing. In addition, academics seek resources to support their personal growth and development to manage the psychological and physical demands of their work (Boğan et al., 2024). While being an academic in Turkey is prestigious, the profession is frequently criticized for unfavorable conditions, such as low pay (Toker, 2011) and demanding workloads (Aydin et al., 2023). Additionally, tenure is granted only to full-time and associate professors, leading to greater employment instability among lower-ranked academics, including research assistants, lecturers, and assistant professors (Tayfur Ekmekci et al., 2021). In this challenging work environment, positive workplace interactions, such as high-quality LMX and CWX, could be crucial support mechanisms, decreasing negative factors in the academic setting. Moreover, positive interactions among academics can create numerous beneficial effects; therefore, encouraging strong interpersonal connections among scholars enhances feelings of affiliation and devotion within the academic community (Makera et al., 2019). By fostering a sense of belonging, support, and recognition among faculty members, these interpersonal dynamics could reduce the negative impacts of workload, pay dissatisfaction, and job insecurity, thereby promoting a more positive work environment. Furthermore, collaborating, sharing ideas, and receiving support from coworkers are becoming increasingly significant in universities. This aligns with this study’s theoretical framework, which asserts that thriving at work, supported by healthy social relationships, is essential for flourishing. A supportive work environment, where employees recognize and respect their peers, was associated with higher levels of thriving (Walumbwa et al., 2020), potentially contributing to a healthier and more productive academic culture in Turkey. Previous studies indicate that the relational mechanisms connecting LMX and CWX to thriving and flourishing have primarily been examined in relatively standardized occupational contexts, such as the IT sector (Bhatti et al., 2022) and the healthcare sector (Di Milia and Jiang, 2024). Noting this limitation, the authors of these studies called for further research in more diverse occupational contexts. Thus, evaluating the nature of these interactions among Turkish scholars presents a unique context for two reasons. First, academia is an independent yet high-pressure environment influenced by publishing requirements and career uncertainty; second, Turkey’s significant power gap might make connections with leaders more critical than those among colleagues. This enables the evaluation of the relative contributions of LMX and CWX under situations distinct from those in previous studies.

### 2.2 Flourishing and workplace interactions

Flourishing is defined as *“the experience of life going well*” (Huppert and So, 2013:838). It is characterized by employees experiencing high levels of wellbeing and functioning at their full potential (Keyes, 2007). It is an increasingly important issue for organizations to address how to attract and retain committed, flourishing employees who contribute to the organization’s success (Bakker and Schaufeli, 2008). Flourishing individuals can develop genuine, trusting connections with others and are willing to enhance their capabilities, evolve, and broaden their horizons (Bakker and Sanz-Vergel, 2013). Those who flourish are well-adjusted and successful, while others feel empty and stagnant, thus believing that their lives lack significance (Keyes, 2002).

Various flourishing models have been suggested; each derived from or centered on distinct aspects of wellbeing. While several studies concentrate on hedonic wellbeing (Diener et al., 1999), others focus on eudaimonic wellbeing (Ryan and Deci, 2001). According to some perspectives (Keyes, 2002), two theoretical views, hedonism and eudaimonia, are the basis for studying wellbeing. Keyes (2002) analyzed the characteristics that lead to flourishing in three dimensions: *“emotional,”* “*psychological,”* and *“social”* wellbeing. Diener et al.'s (2010) flourishing conceptualization includes various elements such as *“having a meaningful life, social relationships, competence, self-respect, engagement, optimism, and making a positive impact on the wellbeing of others.”* Diener et al.’s (2010) perspective on assessing flourishing is among the most widely used and contains both hedonic and eudaimonic dimensions of wellbeing (Huppert and So, 2013). As high levels of flourishing mean significant experiences, researchers have shown that flourishing reduces some risks to both physical and mental health, such as anxiety (Hirshberg et al., 2022) and depression. Within an organizational context, higher levels of flourishing are associated with various positive outcomes, including prosocial attitudes (Hirshberg et al., 2022), contextual performance and creativity (Demerouti et al., 2015), lower absenteeism, and turnover intention (Keyes, 2007). These findings point out the importance of creating supportive organizational environments that foster flourishing.

Although flourishing is closely related to several wellbeing constructs, it is conceptually distinct from them, and clarifying these distinctions is essential to justify its selection as the outcome variable in the present study. Rather than serving as a synonym for wellbeing, flourishing represents a broader, more comprehensive form of optimal psychological functioning. Subjective wellbeing is primarily defined in hedonic terms, encompassing life satisfaction and the balance of positive and negative affect (Diener et al., 1999). In contrast, flourishing integrates both hedonic and eudaimonic dimensions of functioning, including meaning, competence, and positive relationships (Keyes, 2002; Ryan and Deci, 2001). Accordingly, flourishing extends beyond mere life satisfaction. Additionally, life satisfaction refers to individuals’ overall cognitive evaluations of their lives (Diener et al., 1985). As such, it captures only one component of flourishing, which incorporates and expands upon this evaluative dimension. This distinction is also evident in the measurement of these constructs. The Flourishing Scale (Diener et al., 2010), employed in the present study, assesses purpose, relationships, engagement, self-esteem, and optimism rather than a single evaluative judgment, thereby capturing a broader construct than the life satisfaction measures used in related studies (e.g., Zhai et al., 2020).

Flourishing also differs from work engagement, which represents a work-specific motivational state directed toward job tasks and confined to the work role (Bakker and Schaufeli, 2008). By contrast, flourishing reflects a broader life-level outcome that extends beyond the workplace. For consistency, the term “flourishing” is used throughout this study to refer to this integrated hedonic and eudaimonic conceptualization, including studies that use the term “psychological flourishing” (e.g., Singh et al., 2019). Taken together, flourishing is a comprehensive indicator of human functioning that can be supported by workplace social resources, such as LMX and CWX, while extending beyond the boundaries of work-related experiences.

A key variable in determining how employees perceive social interactions at work is their level of LMX. LMX refers to the quality of the supervisor-subordinate social exchange relationship (Dienesch and Liden, 1986). Leaders develop a distinct level of relationship with each employee rather than an average level of relationship with all employees (Graen and Uhl-Bien, 1995). High-quality LMX relations are associated with trust, honesty, interaction, support, loyalty, and respect (Martin et al., 2018). Employees will experience a greater sense of safety and acceptance within the organization thanks to high-quality LMX (Okros and Virga, 2023). On the other hand, relationships characterized by low-quality LMX are limited to fulfilling specific job responsibilities and lack the extensive depth shown in high-quality LMX interactions (Martin et al., 2018).

High-quality LMX is associated with many organizational and individual outcomes, such as decreased anxiety (Fisher et al., 2016), increased empowerment (Kim and George, 2005), and increased thriving (Bhatti et al., 2022). Moreover, leaders enhance employee growth by emphasizing employees’ talents and acknowledging their efforts. Thus, employees can develop trusting relationship with the leader in the workplace (Ramdas and Patrick, 2019). According to SET, trust-based relationships reduce uncertainty and confusion in the workplace, providing employees with a sense of assurance and predictability. In high-quality LMX relationships, employees are more inclined to participate in social interactions that offer essential assets, including knowledge, support, and guidance (Naim and Ozyilmaz, 2023). This mutually beneficial relationship improves employees’ capacity to handle job-related duties while promoting personal and professional growth, crucial components of thriving. Additionally, LMX promotes autonomy and competence, which are fundamental components of SDT. Leaders develop employee competency by providing guidance and constructive criticism, helping employees feel competent in completing their duties.

Furthermore, prior studies showed that supportive and trusted relationships between leaders and employees, engaging and demanding professional responsibilities, and strong relationships with coworkers significantly affect the state of thriving (e.g., Peethambaran and Naim, 2023). Thus, by developing trust, providing relational resources, and minimizing ambiguity, high-quality LMX interactions can create the essential conditions for flourishing. In this context, the following hypothesis was proposed.

*H1*: LMX is positively related to flourishing.

The employee–coworker relationship is characterized by mutual trust and exchange, whereas the relationship between a leader and their subordinates primarily focuses on authority and commands. Exchange interactions can occur between employees and supervisors, but genuine social exchanges of a more intimate and personal nature are more likely among coworkers (McCarthy et al., 2016). CWX is defined as the quality of relationships among employees and coworkers in the same department (Sherony and Green, 2002). Employees who interact with coworkers often experience pleasant, meaningful exchanges that foster strong interpersonal relationships (Kim et al., 2023). Although individuals may find it difficult to express their ideas and concerns to superiors, they tend to be more open with coworkers. Consequently, individuals often seek assistance from coworkers rather than supervisors to navigate work challenges, which enhances wellbeing by effectively managing these challenges (McCarthy et al., 2016).

In the context of SET, CWX is characterized by reciprocal social interactions that prioritize mutual support, trust, and resource exchange. CWX has been associated with several positive outcomes, including enhanced psychological flourishing and a positive working environment (Singh et al., 2019). Additionally, it has been linked to the reduction of work-related stress (Halbesleben, 2012) and workplace anxiety (McCarthy et al., 2016). CWX enhances wellbeing by fulfilling the fundamental psychological need for relatedness (Deci and Ryan, 2012). Consequently, flourishing, which includes wellbeing, is more likely when employees perceive support and appreciation from their coworkers. Given these positive aspects, employees can achieve significant personal and professional growth in the workplace through high-quality CWX. Consequently, the following hypothesis was proposed:

*H2*: CWX is positively related to flourishing.

### 2.3 The mediating role of thriving at work

As people increasingly focus on their careers, the importance of excelling at work has grown. Spreitzer et al. (2005) defined the concept of thriving by distinguishing it from similar concepts and explaining how context influences individuals’ experiences of thriving. Thriving at work reflects an individual’s positive wellbeing, active engagement, and achievement in their work environment (Kleine et al., 2019). It involves two key aspects, including a sense of learning and vitality that enables individuals to prosper in their jobs (Porath et al., 2012). Learning involves acquiring and applying knowledge or skills, while vitality is the positive sensation of having energy. According to Spreitzer et al. (2005), thriving is a temporary emotional state, not a permanent trait that indicates personal growth and development. Moreover, thriving is not only related to reducing stress; it also requires enhancing positive individual, relational, and environmental factors. Contrary to traditional views that success requires overcoming challenges, thriving can occur without significant obstacles (Kleine et al., 2019). Thriving at work is primarily associated with positive relationships and various employee outcomes, including job satisfaction, career development, and general health (Porath et al., 2012).

Self-determination theory explains how high-quality LMX promotes thriving at work by fulfilling the fundamental psychological demands for autonomy, competence, and relatedness (Deci and Ryan, 2012). Leaders who succeed in establishing high-quality LMX relationships are inclined to offer more support and appreciation to their subordinates. Positive interactions with leaders may improve an individual’s perception of achievement and vitality by creating a work atmosphere that promotes mutual respect and trust between supervisors and subordinates (Kleine et al., 2019). Furthermore, because learning requires contact with others for new information and expertise, LMX influences employees’ involvement in learning endeavors (Bezuijen et al., 2010). Moreover, leaders who create opportunities for development and growth enable employees to enhance their abilities, improve their job performance, and subsequently experience higher levels of vitality and learning (Xu et al., 2017). Previous research indicates that leaders who assist their subordinates can enhance their competency, and the quality of LMX can significantly influence thriving at work (Li, 2015). Furthermore, high-quality LMX positively influences workplace productivity and job enrichment, hence increasing employee wellbeing (Zhang et al., 2022). Additionally, LMX has a significant impact on vitality by praising and empowering employees and providing them with feedback (Wu et al., 2023). Therefore, drawing on theoretical assumptions and empirical evidence, the following hypothesis was proposed.

*H3*: LMX is positively related to thriving at work.

As Ryan and Deci (2000) proposed, the inherent inclination for psychological development indicates that thriving individuals actively contribute to forming their social surroundings. As active participants, thriving individuals strive to cultivate high-quality relationships with their leaders and coworkers. They also try to enhance their knowledge and actively pursue chances for ongoing growth (Spreitzer et al., 2005). Additionally, according to SET, relational identification emerges when individuals have high-quality exchange connections with their coworkers. Moreover, employees’ interactions with coworkers significantly impact their work experiences, personal connections, and attitudes toward work (Takeuchi et al., 2011). According to earlier studies, employees report higher vitality levels when they get social support from coworkers (Niessen et al., 2012). In other words, CWX could significantly impact an employee’s workplace experience. Studies indicate that individuals who perceive increased support from colleagues exhibit enhanced thriving at work (Kleine et al., 2019). Given that mutual support among coworkers could improve their positive mental states, such emotions might be beneficial to them. This could improve coworker interactions and increase their willingness to pursue ongoing learning and development in the workplace (Wu et al., 2023). Conversely, the absence of such support could result in employees feeling anxious, isolated, and eventually less motivated. In light of these facts, CWX is considered an essential element for thriving in the workplace, parallel to SDT’s emphasis on relatedness and SET’s focus on the reciprocal dynamics of high-quality workplace relationships. Thus, it was hypothesized:

*H4*: CWX is positively related to thriving at work.

Flourishing and thriving at work are often confused, yet they represent distinct conceptual constructs (Manfreda et al., 2025). This distinction can be illustrated through the botanical metaphor proposed by Lomas et al. (2023), whereby thriving reflects the vigorous growth of an individual plant, whereas flourishing occurs only when both the plant and the wider garden that supports it are doing well. Accordingly, employees may thrive within their work roles by experiencing learning and vitality without necessarily flourishing, because flourishing requires positive functioning across multiple life domains.

Thriving at work represents a feeling of vitality and learning experienced in the workplace (Spreitzer et al., 2005). Furthermore, it has been distinguished from flourishing and subjective wellbeing since it emphasizes vitality and learning derived from the workplace (Carmeli and Spreitzer, 2008). According to Spreitzer et al. (2005), thriving at work helps individuals focus on their goals and adapt to changing circumstances, leading to positive mental and physical health. In parallel with this perspective, three aspects of wellbeing, namely emotional, psychological, and social, were found to influence thriving at work (Lezar and van der Walt, 2023). Thriving at work is positively related to variables such as psychological capital, positive affect, and work engagement, which could influence flourishing (Kim et al., 2023; Kleine et al., 2019). Additionally, since thriving at work relies on continuous learning and development, the skills and competencies employees acquire within the organization can help them throughout their careers, even if they later work for a different organization.

Flourishing, on the other hand, serves as a more comprehensive measure of psychological wellbeing (Diener et al., 2010). It includes several dimensions of socio-psychological wellbeing, such as satisfying relationships, self-esteem, a feeling of purpose, and optimism. Flourishing pertains to the condition in which an individual operates effectively in both psychological and social dimensions (Keyes and Haidt, 2003). It encompasses both hedonic and eudaimonic aspects and is considered a broader approach to evaluating individuals’ wellbeing than the subjective measure of wellbeing typically referred to as happiness (Mesurado et al., 2021). Thus, flourishing involves a more general assessment of an individual’s wellbeing, encompassing satisfaction in areas such as life, job, family, and health; nevertheless, both concepts incorporate positive elements of human functioning (Spreitzer et al., 2005). On the other hand, thriving at work refers to a distinct and focused positive mental state characterized by energy and growth at work. This conceptual distinction indicates that thriving at work is closely linked to personal growth, which subsequently enhances overall life contentment. Experiencing a sense of vitality and learning enhances individuals’ ability to withstand physical challenges and disease and improve wellbeing (Walumbwa et al., 2020). Prior research has shown that thriving at work is linked to broader life-related variables. These include wellbeing (Okros and Virga, 2023), life satisfaction (Zhai et al., 2020), and subjective health (Kleine et al., 2019; Walumbwa et al., 2018). Therefore, as previous empirical findings have also indicated, thriving at work, as a construct distinct from flourishing, is considered a potential antecedent of flourishing. Thus, it was hypothesized:

*H5*: Thriving at work is positively related to flourishing.

Given that factors such as engaging in decision-making, participating in open information sharing, receiving feedback to improve performance, and feeling supported in easing work challenges contribute to personal development and energy in the workplace, it is believed that there is a favorable relationship between LMX and flourishing (Kleine et al., 2019). This perspective is strengthened by research demonstrating that high-quality LMX makes workers feel more empowered, volitional, and autonomous at work (Gómez and Rosen, 2001). Thriving employees are thought to be more inclined to experience a sense of empowerment and support, resulting in elevated levels of wellbeing and personal development, which are fundamental aspects of flourishing. For instance, in a study conducted with Chinese supervisor-employee dyads, thriving was found to mediate the effects of LMX on affective commitment and job performance (Li, 2015). Bhatti et al. (2022) examined the mediating role of thriving at work in the relationship between LMX and creative performance. Di Milia and Jiang (2024) also found that LMX increased work-life balance through thriving at work. Thus, empirical findings suggest that LMX may influence both work and nonwork contexts through its effects on thriving at work. Moreover, while Imran et al. (2020) examined thriving at work and flourishing as two parallel mediators in the relationship between perceived organizational support and work engagement, the present study emphasizes positioning thriving at work not merely as a mechanism leading to work outcomes but as a developmental step that contributes to flourishing. Drawing on theoretical and empirical support, employees are more likely to thrive when they experience growth in learning and feel energized in their current roles, which in turn extends to the optimal configuration of wellbeing. Thus, the following hypothesis was proposed:

*H6*: The positive relationship between LMX and flourishing is mediated by thriving at work.

Establishing strong and positive workplace connections could be energizing, as it is critical to connect attentive and mindful interactions with vitality (Heaphy and Dutton, 2008). Previous studies show that attentive relationships correlate with personal growth and success (Niessen et al., 2012). Employees improve their skills through exchanges with their peers. However, limited research exists on how LMX and CWX, combined, affect flourishing. Previous studies have demonstrated a positive correlation between being attentive in relationships and experiencing personal growth and success (Niessen et al., 2012). Thus, employees could enhance their skills and knowledge by exchanging with their peers (Paterson et al., 2014). Although some studies explore the combined influence of LMX and CWX on various employee outcomes (Zhang et al., 2022), there is limited knowledge about how these combined effects play a role in flourishing. Similarly, Kleine et al. (2019) found that supportive coworker behavior positively influences thriving at work. Walumbwa et al. (2020) suggested that, in accordance with SET, relational resources created during employment influence individuals through situational processes, such as increased organizational and group-level identification, thereby increasing thriving at work. Additionally, Zhai et al. (2020) showed that thriving at work moderates the links between workplace support and life satisfaction. Positive interactions with coworkers increase employees’ capacity to handle stress and conflicts (McCarthy et al., 2016). Based on that idea, flourishing could increase as CWX develops, and employees could improve their capacity to handle interpersonal and organizational challenges. SET asserts that mutual trust and support create the foundation of high-quality relationships. Singh et al. (2019) found that coworker exchange was positively related to psychological flourishing. Thus, supportive coworker relationships enable employees to grow, develop, and feel fulfilled in their roles. Coworkers who provide feedback, share knowledge, and assist each other in problem-solving can promote skill development and reinforce a sense of competence. Thus, drawing upon theoretical and empirical knowledge, the following hypothesis was proposed:

*H7*: The positive relationship between CWX and flourishing is mediated by thriving at work.

**Figure 1:**
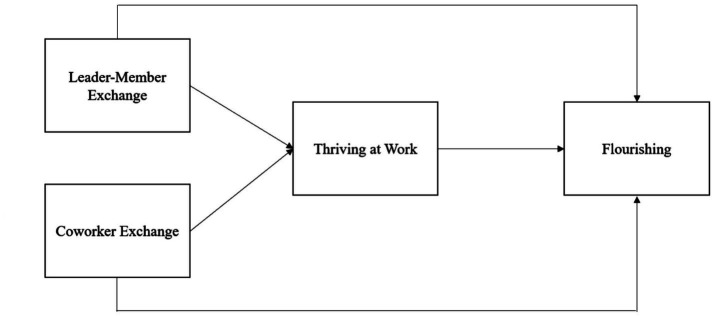
Research model.

Figure 1 presents the conceptual model illustrating the relationships among the study variables and the proposed hypotheses.

## 3 Materials and methods

### 3.1 Participants

This study employed a convenience sampling technique to collect cross-sectional data from academics working at public and private institutions in Turkey. The research sample comprised 418 academics. The participants were recruited using an integrated strategy that involved direct contact with individuals at different universities and the use of professional networks. The data were collected through both online and paper-based questionnaire administration methods to reach a broader range of participants. The online survey form was prepared using Google Forms and distributed to potential participants through personal contacts, meetings, and social media channels. The survey instructions clearly stated that participation was voluntary. In addition, printed versions of the questionnaire were administered face-to-face by the researchers to academics who voluntarily agreed to participate in the study. The participants completed the questionnaires voluntarily in both data collection procedures. The study primarily focused on examining the relationships among the main research variables rather than conducting comparative analyses based on demographic characteristics. Therefore, demographic variables were collected only to provide general information about the sample. In this context, age, gender, sector, and organizational tenure were included as descriptive variables. Regarding gender, 66% (*n* = 276) of the participants were female, and 34% (*n* = 142) were male. The age distribution was as follows: 32.3% (*n* = 135) were between the ages of 24 and 30; 25.8% (*n* = 108) were between 31 and 37; 22% (*n* = 92) were between 38 and 44; and 19.9% (*n* = 83) were over 45 years old. In terms of sector distribution, 61.2% of the participants (*n* = 256) were employed in the public sector, while 38.8% (*n* = 162) were in the private sector. Turkish higher education is highly centralized, with the Council of Higher Education (YÖK) governing faculty appointments, promotions, and curriculum standards for both public and private institutions (Yasa and Aksoy, 2025). Accordingly, although certain institutional differences exist between public and private universities, in terms of administrative structure and working conditions, academics in both sectors share a common regulatory framework. This framework prescribes the same appointment and qualification criteria and the same core academic responsibilities, including teaching, research, and administrative duties. Therefore, participants from public and private universities were evaluated together within the scope of the study.

Regarding organizational tenure, 34.4% of the participants had 1–5 years of tenure (*n* = 144); 19.9% had 6–10 years (*n* = 83); 13.2% had 11–15 years (*n* = 55); 10.0% had 16–20 years (*n* = 42), and 22.5% had more than 20 years (*n* = 94) (Table 1).

**Table 1:** Demographic characteristics of the sample.

Variable	Category	*n*	%
Gender	Female	276	66.0
	Male	142	34.0
Age	24–30 years	135	32.3
	31–37 years	108	25.8
	38–44 years	92	22.0
	45 years and above	83	19.9
Sector	Public	256	61.2
	Private	162	38.8
Organizational tenure	1–5 years	144	34.4
	6–10 years	83	19.9
	11–15 years	55	13.2
	16–20 years	42	10.0
	More than 20 years	94	22.5

*N* = 418. Source: Table by authors.

### 3.2 Procedure

The data collection tool included self-report measures and demographic questions. Before starting the survey, participants gave formal consent and were informed of their right to withdraw. Data confidentiality and academic use were assured. The study received ethical approval from the co-author’s institution. AMOS (IBM SPSS Amos) was used to evaluate the research model. After establishing the measurement model, structural equation modeling (SEM) was employed to assess the hypotheses. The statistical fit measures and standardized path coefficients were reported. The structural model’s fit was evaluated using the χ2 statistic, the root mean square error of approximation (RMSEA), the standardized root-mean-square residual (SRMR), the comparative fit index (CFI), and the Tucker–Lewis Index (TLI).

### 3.3 Measures

All measures used in this study were translated from English to Turkish and then back-translated, following Brislin (1980).

*Leader-member exchange*. Graen and Uhl-Bien’s (1995) “Leader-Member Exchange (LMX) Scale,” which consists of seven items, was employed to capture the respondents’ perceptions of LMX. The participants rated items on a five-point Likert scale (1 = “strongly disagree,” 5 = “strongly agree”), with higher scores indicating higher LMX. An example item is as follows: *“I have enough confidence in my leader, and I support all his/her decisions.”*

*Coworker exchange*. CWX was assessed using a scale adapted by Sherony and Green (2002) from the LMX scale (Graen and Uhl-Bien, 1995). The scale consists of six items, with a sample item “*My coworkers understand my job problems and needs.”* Each item is rated on a 5-point Likert scale that ranges from 1 (strongly disagree) to 5 (strongly agree).

*Thriving at work*. The 10-item “Thriving at Work” measure developed by Porath et al. (2012) assessed levels of thriving at work. This measure includes five items on learning and five on vitality. A sample item for learning is “*At work, I continue to learn more and more as time goes by.”* A sample item for vitality is “*At work, I feel alive and vital.”*

*Flourishing*. The 8-item “Flourishing Scale” (Diener et al., 2010) was utilized to measure the participants’ levels of flourishing. The scale ranges from 1 (strongly disagree) to 5 (strongly agree). A sample item is *“I lead a purposeful and meaningful life.”*

## 4 Results

### 4.1 Descriptive statistics

The descriptive statistical data from the measuring instruments are shown in Table 2, including skewness and kurtosis values, bivariate correlations, means, standard deviations, composite reliabilities, and average variance extracted (AVE) values. The results presented in Table 2 indicate significant positive correlations between LMX and CWX (*r* = 0.31, *p* < 0.01), LMX and thriving at work (*r* = 0.38, *p* < 0.01), and LMX and flourishing (*r* = 0.31, *p* < 0.01). Furthermore, there is a positive correlation between CWX and thriving at work (*r* = 0.16, *p* < 0.01), as well as between CWX and flourishing (*r* = 0.35, *p* < 0.01). Besides, thriving at work and flourishing are positively correlated (*r* = 0.58, *p* < 0.01). Hence, it was determined that all variables had substantial and favorable correlations, indicating that H1 and H2 were supported. The composite reliability values of the scales are as follows: 0.94 for LMX, 0.93 for CWX, 0.88 for thriving at work, and 0.84 for flourishing. These values indicate acceptable reliability, as they exceed the threshold of 0.70 (Hair et al., 2019). The scales’ average variance extracted values are 0.68, 0.68, 0.56, and 0.50, respectively. Since these values exceed the required criterion of 0.50, as proposed by Fornell and Larcker (1981), they are considered acceptable. The diagonal value for LMX exceeds all its correlations with other constructs, indicating that LMX explains more variance with its indicators than any other latent variable. The same pattern is observed in CWX, which also shows a greater diagonal value than its inter-construct correlations. Similarly, thriving at work and flourishing have diagonal values that are above their respective correlations with other variables. These results, taken together, suggest that all constructs meet the Fornell–Larcker criterion for discriminant validity.

**Table 2:** Descriptives.

Variables	Mean	SD	1. LMX	2. CWX	3. TW	4. FL	CR	AVE	Skewness	Kurtosis
1. LMX	3.26	1.09	0.83				0.94	0.68	−0.331	−0.696
2. CWX	3.58	0.92	0.31**	0.82			0.93	0.68	−0.729	0.289
3. TW	3.79	0.85	0.38**	0.16**	0.75		0.88	0.56	−0.775	0.448
4. FL	4.03	0.64	0.31**	0.35**	0.58**	0.70	0.84	0.50	−1.013	2.50

*N* = 418. Values on the diagonal represent the square roots of the AVE for each construct. LMX: Leader-member exchange, CWX: Coworker exchange, CR: Composite Reliability, AVE: Average Variance Extracted **p < 0.01. Source: Table by the authors.

### 4.2 Measurement model

To evaluate the measurement tools, the method proposed by Bagozzi and Edwards (1998) was followed. Confirmatory factor analysis (CFA) using the maximum likelihood method was conducted in SPSS AMOS to assess multiple measurement models. Five alternative confirmatory factor analyses (CFAs) were conducted to evaluate the distinctiveness of the study variables (Table 3). The original model consisted of a four-factor structure, where each research variable was treated as an independent factor. Two alternative three-factor models were examined alongside the four-factor model for comparison. The first three-factor approach combined LMX and CWX into a single factor, whereas thriving at work and flourishing remained separate factors. The second three-factor approach collapsed thriving at work and flourishing into a single factor, whereas LMX and CWX were treated as distinct factors. The two-factor model combined LMX and CWX into a single component, while thriving at work and flourishing were collapsed into another factor. Additionally, to assess the potential risk of common method bias, Harman’s single-factor test was conducted by loading all measurement items onto a single latent factor in AMOS. The model fit was evaluated using several statistical metrics, including the χ2 statistic, normed chi-square (χ2/df), RMSEA, SRMR, and CFI. The RMSEA and SRMR values below 0.05 indicated a satisfactory fit, as Browne and Cudeck (1992) stated. In addition, CFI values that were equal to or better than 0.90 were considered acceptable.

**Table 3:** Comparison of the alternative measurement models.

Model	χ^2^	df	χ^2^/df	*p*	RMSEA	SRMR	CFI	TLI
1. Four-factor model	912.125	264	3.455	0.000	0.07	0.07	0.92	0.90
2. Three-factor model^a^	1641.780	272	6.036	0.000	0.11	0.10	0.82	0.78
3. Three-factor model^b^	2849.111	272	10.475	0.000	0.15	0.15	0.66	0.58
4. Two-factor model	3356.760	274	12.251	0.000	0.16	0.16	0.60	0.50
5. Single-factor model	4780.846	275	17.385	0.000	0.19	0.19	0.41	0.27

*N* = 418. RMSEA = root mean square error of approximation; SRMR = standardized root mean square residual; CFI = comparative fit index; TLI = Tucker-Lewis index. Source: Table by the authors.

^a^ LMX and CWX were combined into a single factor, while thriving at work and flourishing were specified as separate factors.

^b^ b Thriving at work and flourishing were combined into a single factor, while LMX and CWX were specified as separate factors.

According to the information presented in Table 3, CFA provided the following results for the four-factor model: χ2(912.125, *N* = 418)/df (264) = 3.455, *p* < 0.001, RMSEA = 0.07; SRMR = 0.07; CFI = 0.92, indicating acceptable model fit. Compared with the four-factor model, the three-factor model^a^ [χ^2^(1641.780, *N* = 418) / df (272) = 6.036, *p* < 0.001, RMSEA = 0.11; SRMR = 0.10; CFI = 0.82] and the three-factor model^b^ [χ^2^(2849.111, *N* = 418) / df (272) = 10.475, *p* < 0.001, RMSEA = 0.15; SRMR = 0.15; CFI = 0.66] showed worse fit indices. In addition, the two-factor model [χ^2^(3356.760, *N* = 418) / df (274) = 12.251, *p* < 0.001, RMSEA = 0.16; SRMR = 0.16; CFI = 0.60] showed worse fit indices than the four-factor model. To assess the potential for common method bias, Harman’s single-factor test was conducted by loading all measurement items onto a single latent factor in AMOS. The single-factor model demonstrated poor fit to the data [χ^2^(4780.846, *N* = 418)/df (275) = 17.385, *p* < 0.001, RMSEA = 0.19; SRMR = 0.19; CFI = 0.41]. Therefore, the four-factor model exhibited better model fit than the other models, suggesting that our measuring instruments had satisfactory validity.

### 4.3 Structural model

Structural equation modeling was employed to test our research model. Table 4 shows the overall structural model results, including direct, indirect, and total effects. As shown in Table 4, the direct effect of LMX on thriving at work (*β* = 0.36, *p* < 0.01) was significant, whereas the direct effect of LMX on flourishing (*β* = 0.02, *p* > 0.01) was not significant. Thus, H3 was supported. The results suggested that CWX had a substantial and statistically significant positive impact on flourishing (*β* = 0.26, *p* < 0.01). However, there was no significant direct influence of CWX on thriving (*β* = 0.04, *p* > 0.01), indicating that H4 was not supported, while H5 was supported.

**Table 4:** SEM results.

Path	DE	95% CI	IE	95% CI	TE	95% CI	
LMX → Thriving	0.36**	[0.19,0.41]			0.38**	[0.18,0.41]	
LMX → Flourishing	0.02	[0.25,0.47]			0.30**	[0.27,0.47]	
CWX → Thriving	0.04	[−0.07,0.18]			0.04	[−0.07,0.18]	
CWX → Flourishing	0.26**	[0.12,0.39]			0.28**	[0.13,0.43]	
Thriving→Flourishing	0.53**	[0.39,0.64]			0.52**	[0.39,0.64]	
LMX → Thriving→Flourishing			0.28**	[0.19,0.37]			
CWX → Thriving→Flourishing			0.03	[−0.04,0.09]			

DE = Direct effect; IE = Indirect effect; TE = Total effect; CI = confidence interval. Model fit indices: χ^2^ = 912.125, df = 264, χ^2^/df = 3.455, CFI = 0.92, TLI = 0.90; SRMR = 0.07, RMSEA = 0.07, *p* = 0.000; *p < 0.05, **p < 0.01. Source: Table by the authors.

To assess indirect effects, 95% bootstrap confidence intervals were computed using 5,000 bootstrap samples. Table 4 shows that the mediator thriving at work had a significant indirect effect on the relationship between LMX and flourishing (*β* = 0.28, *p* < 0.01). This finding implied that there was no direct link between LMX and flourishing. Instead, the connection between LMX and flourishing was mediated by the level of thriving at work. Hence, H6 was supported. Additionally, the indirect effects of thriving at work on the relationship between CWX and flourishing (*β* = 0.03, *p* < 0.01) were not significant. Thus, CWX directly affected flourishing, but not through thriving at work. Therefore, H7 was rejected.

**Figure 2:**
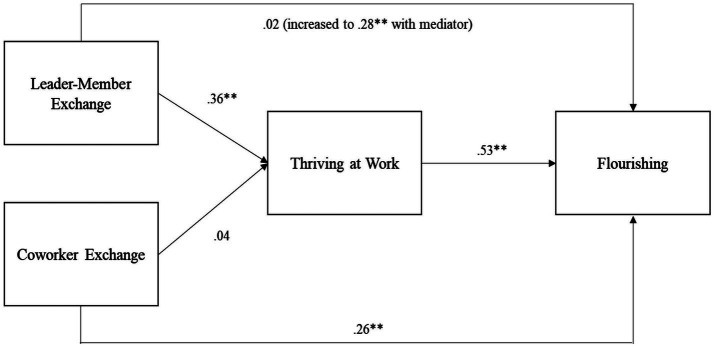
Research model with SEM results.

Figure 2 presents the results of the structural equation modeling (SEM), which graphically illustrates the relationships among the variables under consideration.

## 5 Discussion

Flourishing is an important topic that needs more research in academic settings since it is closely linked to greater performance (Demerouti et al., 2015), decreased absenteeism, and lower turnover intention (Keyes, 2007). Flourishing is especially salient among university academics, whose capacity to flourish is shaped by sector-specific demands such as work overload and limited career certainty (Stratford et al., 2024). Several studies have recognized LMX and CWX as important interpersonal assets in the workplace (Walumbwa et al., 2020; Zhai et al., 2020). This study distinguishes itself by examining the combined impact of LMX and CWX on fostering flourishing through thriving at work among academics.

The results showed that LMX does not have a direct effect on flourishing. However, it was found that thriving at work mediates the relationship between LMX and flourishing, indicating an indirect link between LMX and flourishing. This finding could stem from the connection between a leader and their subordinates (e.g., a department chair or senior colleague), which may not immediately affect flourishing, since flourishing extends beyond the workplace context. LMX is fundamentally a construct that pertains specifically to work, whereas flourishing is more comprehensive and spans other aspects of an academic’s life. Accordingly, previous studies have suggested potential mediators between leadership behavior and its positive impact on happiness, wellbeing, and flourishing, such as trust (Ramdas and Patrick, 2019) and need satisfaction (Dose et al., 2019). This perspective is also consistent with previous LMX research, which found that the effects of high-quality leader-member relationships are frequently explained through underlying psychological mechanisms rather than occurring directly. For instance, Aggarwal et al. (2020) showed that psychological empowerment mediates the relationship between LMX and positive work outcomes, whereas Götz et al. (2020) found that organizational identification explains how LMX promotes positive workplace behaviors. Our findings also support previous evidence emphasizing the importance of LMX in explaining employee outcomes (Shkoler et al., 2021). These findings show that the indirect effect of thriving suggests that the benefits of high-quality leader-member interactions may not always be immediately visible in the workplace but can eventually spread to other domains of life and contribute to flourishing. From the perspective of SET, our findings suggest that high-quality leader-member relationships provide valuable relational resources that support thriving at work, thereby contributing to employees’ development and flourishing.

In this study, since CWX did not directly affect thriving at work, the assumption that thriving at work mediates the relationship between CWX and flourishing was rejected. The distinct relational dynamics of CWX compared to LMX could explain the absence of a direct influence of CWX on thriving at work, as opposed to its impact on flourishing in this study. In particular, while thriving at work encompasses academic growth and vitality, colleagues may not thrive in contexts where organizational policies and procedures focused on development are more sensitive to leader-member interactions. However, it was found that CWX has a direct effect on flourishing. According to Li and Hung's (2009) study, LMX is a more effective predictor of task performance than CWX, while CWX is a stronger predictor of organizational citizenship behavior. As organizational citizenship behavior arises from voluntary actions, these findings could be interpreted as CWX directly impacting social and emotional dimensions of work-life and also directly affecting academics’ wellbeing. In contrast to the relationship patterns observed in LMX, CWX encompasses a broader array of connections that extend beyond the professional domain and into personal support and friendship. Additionally, CWX could assist academics in developing and maintaining workplace friendships, leading to a decrease in psychological stress experienced by academics and an improvement in their psychological wellbeing (Halbesleben, 2012). Given the limited influence of coworker interactions on academics learning and vitality processes, it is possible that they did not exert a direct effect on thriving at work. The absence of a direct impact hindered the analysis of the mediating effect. Nonetheless, given the characteristics of interpersonal relationships that can transcend professional boundaries, it is likely that CWX directly influences flourishing.

As with many other countries, building relational resources through interactions with others could play a major role in developing a strong academic community in Turkey’s academic environment. Academics who thrive could experience increased research output, improved teaching evaluations, greater job satisfaction, and enhanced wellbeing. According to the findings, LMX positively affects thriving at work. The results emphasize the significance of interpersonal assets in the professional setting, indicating that promoting favorable social interactions at Turkish institutions and in several academic contexts may result in more energetic academics (Zaimoğlu and Dağtaş, 2025). This assumption aligns with the SET as it emphasizes that the quality of social interactions in the academic environment can result in meaningful social connections, enhancing overall wellbeing (González-Navarro et al., 2019). Conversely, the findings indicate that CWX does not significantly affect thriving at work. One possible reason could be that exchanges among colleagues may have less impact than those of leaders or supervisors. Additionally, some interpersonal characteristics could affect CWX. Considering the Turkish academic environment is generally characterized by high hierarchical structures, it is possible that supervisors’ encouragement and comments often play a more crucial role than peer connections in fostering an academics sense of thriving in the study context. In the Turkish academic context, where the supervisor holds a pivotal role in decisions about working conditions, resource allocation, and promotion (Tayfur Ekmekci et al., 2021), the leader-member relationship appears to be the principal channel through which academics obtain the guidance, recognition, and developmental opportunities that fuel learning and vitality. This helps explain why LMX, rather than CWX, emerged as the significant predictor of thriving at work, and why the benefits of high-quality leadership reach flourishing indirectly, through thriving.

Turkey was ranked among nations with high levels of “collectivism,” “uncertainty avoidance,” and “power distance” in Hofstede et al.'s (2010) cultural analysis. Due to the high-power distance in the cultural context, academic relationships are similar to master-apprentice dynamics. Instructions from a direct supervisor regarding work conditions significantly influence the workplace, with the supervisor playing a pivotal role in decisions related to working conditions and the promotion of academic staff. Moreover, collaboration among researchers with varied strengths is encouraged in collectivist cultures that prioritize group cohesion. However, CWX is not significantly correlated with thriving at work in this study. Although Turkey is classified as a collectivist society, its collectivist tendencies have declined, indicating that it is progressing toward a more individualized society (Hofstede et al., 2010). Similarly, contemporary modifications in academic promotion standards have mandated that scholars conduct academic research independently instead of collaborating as a group. Furthermore, the competitive process for securing academic appointments at universities may foster a culture in which individual ambitions are emphasized over collective aspirations. Under such conditions, colleagues may increasingly be experienced as competitors for scarce positions and promotions rather than as sources of developmental support, offering a further, context-specific explanation for why coworker exchange did not translate into thriving at work in this study.

The results of this study showed that CWX and flourishing were positively related. One significant point is that positive coworker interactions can compensate for the demanding nature of academic work settings, which expose academics to heavy workloads, limited pay, and job insecurity. These pressures are particularly pronounced for lower-ranked academics in Turkish higher education and are linked to burnout and diminished wellbeing (Toker, 2011; Tayfur Ekmekci et al., 2021; Marck et al., 2024; Tepe et al., 2025). Collaborative sharing of ideas and support can serve as a coping method to help academics deal with the inherent stresses of research, teaching, and administrative responsibilities. Positive connections with coworkers can help fill in the gaps by exchanging information, offering practical help, or providing emotional support. Overall, this collaborative support system helps academics become more resilient when facing difficulties, positively impacting their flourishing. Taken together, in the Turkish academic context, coworker exchange appears to enhance flourishing chiefly through socio-emotional support and friendship that buffer these pressures, rather than through the work-based learning and vitality that constitute thriving; the latter, under the strong hierarchy of Turkish academia, seems to be driven more by the supervisor relationship.

### 5.1 Theoretical implications

The present research examined the connections among relational resources, personal growth, and broader outcomes in life. For instance, Zhai et al. (2020) showed that supervisor support and coworker support influence life satisfaction through thriving at work. Kleine et al. (2019) also highlighted that relational resources, such as support, are significant determinants of thriving at work. Similarly, Kleine et al. (2023) investigated the effects of supervisor and coworker support on thriving at work, as well as the following effects on employee wellbeing. However, while support reflects behavior-based acts of providing resources and helping one another in the workplace, the quality of exchange in relationships focuses on more fundamental relational dynamics such as trust and mutuality. From this viewpoint, support is primarily behavior-oriented, whereas exchange is more specific to relationships, since it is based on SET. Walumbwa et al. (2020) also highlighted that while leader and coworker perspectives have often been examined separately, considering them together provides a broader understanding of various outcomes. The findings of this study extend SET by highlighting how LMX and CWX contribute to flourishing, suggesting that the quality of workplace relationships functions through distinct mechanisms to affect flourishing. LMX has a significant indirect impact on flourishing by improving personal growth.

This study demonstrates that thriving at work is a mediating element, revealing the psychological process by which positive interactions lead to flourishing. Since thriving at work refers to being energetically engaged and continuously developing, it could be achieved through positive interactions with leaders and coworkers that provide fulfillment and support (Colbert et al., 2016). This supports the idea that leader-member relationships might create an environment where academics feel valued and autonomous, consequently meeting their fundamental psychological needs, as SDT proposes (Deci and Ryan, 2012). In addition, the direct impact of CWX on flourishing highlights the importance of interactions with peers in fulfilling academics’ need for connection, further parallel to SDT’s assumptions. This discovery clarifies the significance of coworker relationships in providing emotional support and fostering a sense of belonging, directly enhancing an academics’ flourishing experience. Among academics specifically, enabling conditions such as supportive mentoring, manageable workloads, and meaningful career development have been identified as key to flourishing (Stratford et al., 2024). The findings of this study underline the significance of fostering hierarchical and collaborative connections inside universities to establish an appropriate academic growth and development environment. Further research could investigate ways to help academics thrive at work to enhance flourishing and increase the positive consequences of interactions at work, as thriving at work appeared to mediate the relationship between LMX and flourishing in the current study.

### 5.2 Practical implications

The results of this study, which focused on the positive impacts of workplace interactions, provide practical insights for academic leaders, such as department chairs, deans, and university administrators, as well as human resource units in higher education, who aim to improve academics’ thriving and overall flourishing. Academic leaders must recognize that a greater number of academics now receive satisfaction from social and developmental relationships and should adopt a more supportive role, encouraging academics with autonomy over their teaching and research to thrive (Liu D. et al., 2021). This is particularly important for early-career academics, including research assistants, lecturers, and assistant professors, who often face precarious contracts, heavy workloads, and burnout, and who benefit from stable conditions, mentoring, and professional development (Marck et al., 2024; Stratford et al., 2024). Furthermore, promoting constructive interactions among colleagues directly enhances flourishing. This may be accomplished by encouraging co-authorship and joint research projects, establishing peer mentoring and recognition schemes for junior academics, and supporting informal scholarly exchange. These initiatives have the potential to reinforce social connections, facilitate collaborative research efforts, and satisfy academics’ need for interpersonal relationships, which is particularly relevant given the collectivist values prevalent in Turkish higher education institutions. To enhance the quality of LMX at higher educational institutions, department chairs and deans should increase the participation of academics in decision-making processes, which can strengthen the sense of belonging.

Certain approaches, such as developing an open and fair departmental culture and promoting extra-role behaviors, could improve collegial relationships, hence facilitating workplace thriving (Wu et al., 2023) and increasing its influence on flourishing. While this study could not establish a direct link between CWX and thriving at work, it is believed that efforts to increase CWX, which is positively associated with flourishing, would yield advantageous outcomes. Promoting favorable colleague relationships in the workplace could be achieved by integrating them into faculty development programs, fostering team-building activities, and implementing institutional incentive systems (Liu D. et al., 2021; Walumbwa et al., 2020). Also, universities could consider revising leader-member dynamics and coworker interactions to enhance workplace thriving. For instance, establishing interdisciplinary boards or discussion groups within the academic context promotes a sense of community and intellectual stimulation by facilitating collaboration and idea-sharing among academics from different fields.

### 5.3 Limitations and future directions

The generalizability of our findings may be limited by the sample and setting from which our data were collected. The study’s participants, from a single cultural background, may not accurately represent the broad global workforce. This constraint implies that the researchers should be careful when applying our findings to other industries or cultural settings, as the dynamics of the workplace and the characteristics of LMX and CWX may change significantly. Second, this study is based on cross-sectional data. Additionally, since the data were acquired solely through survey research, there is a potential for common method bias to some extent (Podsakoff et al., 2012). Also, the data were collected from a single source at a single point in time, which constitutes another limitation of this study. Future studies might employ longitudinal designs to gain a more thorough understanding of causal pathways and dynamic interactions. This study focused solely on thriving as a mediator in workplace interactions, overlooking other potential mediators and moderators that could influence flourishing. Future research should explore variables such as job insecurity and perceived workload, both salient features of academic work, as mediators, and factors such as psychological capital, proactive personality, and resilience as moderators. Also, LMX and CWX could be linked to work engagement together with thriving at work (Walumbwa et al., 2020) and academics’ flourishing.

## Data Availability

The study data are available from the corresponding author upon any request.
